# Copper extraction and phytotoxicity of organic acid leached mine tailings in *Brassica napus*

**DOI:** 10.1007/s10653-026-03188-7

**Published:** 2026-04-14

**Authors:** Vinicius H. De Oliveira, Sarah Duddigan, James Symons, Michael J. Whelan, Vimalnath Selvaraj, Andrew P. Abbott, Rich Crane, Gawen R. T. Jenkin, Mark Tibbett

**Affiliations:** 1 Department of Sustainable Land Management & Soil Research Centre, School of Agriculture Policy and Development, University of Reading, Reading, UK; 2 Department of Geography & Environmental Science, University of Reading, Reading, UK; 3 Centre for Sustainable Resource Extraction, University of Leicester, Leicester, UK; 4 School of Geography, Geology and the Environment, University of Leicester, Leicester, UK; 5 School of Chemistry, University of Leicester, Leicester, UK; 6 Camborne School of Mines, Department of Earth and Environmental Sciences, University of Exeter, Exeter, UK

**Keywords:** Citrate, Ecotoxicity, Heavy metals, LMWOAs, Remediation

## Abstract

Mine tailings pose environmental hazards but can also contain economically valuable metals like copper (Cu). Organic solvents, particularly low molecular weight organic acids (LMWOAs), are natural and biodegradable acids with great potential for Cu removal and tailings remediation. We evaluated the potential of citric, malic, maleic, malonic and lactic acids (concentration of 1 M) in extracting Cu from legacy Cu-tailings, as well as their phytotoxicity risk. *Brassica napus* was grown in tailings leached by LMWOAs at three dilutions (1/4, 1/16, 1/128) to simulate solvent biodegradation or dilution by rainfall. Plant height, biomass, chlorophyll, shoot green cover and leachate metal concentrations (Al, Ca, Cu, Fe, K, Mg, Mn, V, Zn) were measured; deionised water was used as a control. At 1/4 dilution, citric, malic, and malonic acids generated the highest Cu concentrations in leachate (625–965 mg L^−1^). This resulted in poor plant growth, likely due to Cu and Al toxicity. At 1/128 dilution, all acids had minimal impact on growth, with plant biomass similar to controls (41 g), though citric acid mobilised more elements at nutritional levels. Increased macronutrient (Ca, K, Mg) availability from diluted acids led to lower root/shoot ratios, suggesting partial alleviation of nutrient deficiencies. Multivariate analyses showed distinct elemental leaching profiles among LMWOAs, that converge with dilution, except for citric acid, which retained discrete patterns even at 1/128 dilution. These findings indicate that citric acid has strong potential for Cu-tailings remediation and rehabilitation, initially extracting high Cu and Al concentrations and supplying essential nutrients as concentrations are diluted.

**Supplementary Information:**

The online version contains supplementary material available at 10.1007/s10653-026-03188-7.

## Introduction

Metalliferous mining is an essential practice for society and, despite emerging opportunities for resource reuse, it continues to serve as the primary source of metals (Conde, 2017; Tibbett, 2024). Copper (Cu) and its alloys are extremely important for construction and transportation industries as well as for electrical and electronic equipment (e.g. decking, panels, train cables, batteries), making Cu mining a widespread industry that has more than tripled in scale in the last 50 years (Pietrzyk & Tora, 2018). Global annual refined copper demand has now reached 27 Mt and is set to further expand to approximately 37 Mt by 2050 (IEA, 2025).

Ore processing after mining generates tailings, a finely-ground waste product that harbours a variable quantity of target and non-target metals. Tailings are typically stored behind earthen dams, known as tailings storage facilities (TSFs) (Adiansyah et al., 2015; Tibbett, 2024). The quantity of tailings generated during the mining process is considerable. For example, processing 100 tonnes of low-grade ore with 0.7% copper would yield only 0.7 tonne of refined Cu, but 99.3 tonnes of tailings. Their accumulation in dams can occupy immense areas of land, impacting the local ecology at landscape scale (Sun et al., 2018). Globally, it is estimated that approximately 200 Gt of tailings are stockpiled across hundreds of thousands of locations, with Cu tailings accounting for roughly 40% of this total (Valenta et al., 2023).

The combination of their immense global footprint, the fact that tailings often contain elevated concentrations of potentially toxic metals within soluble minerals, their susceptibility to physical instability, and their potential to generate airborne dust means that they pose profound global-scale environmental and human health risks (Degani et al., 2022; DeJong et al., 2015; Sun et al., 2018; Tibbett et al., 2025). Thus, tailings pose a major liability for mining companies and require constant management.

On the other hand, tailings can represent a secondary source of metals, if these can be recovered. Processes such as hydrometallurgy and solvometallurgy have been proposed as potential technologies for metal recovery (Binnemans & Jones, 2017). Considering their hazardous potential, high levels of heavy metals, lack of structure, nutrients and organic matter to sustain vegetation (Sun et al., 2018; Tibbett, 2024), the further extraction of metals from mine tailings has been regarded as a potential strategy for remediation and rehabilitation (De Oliveira et al., 2024; Jenkin et al., 2024; Li et al., 2025). The extraction of metals from ores and metallic substrates by organic and inorganic solvents has been considered as a promising strategy to enhance metal recovery while decontaminating mine tailings, which enhances their potential for the development of viable ecosystems (Binnemans & Jones, 2017).

Solvents based on organic acids (low molecular weight organic acids, LMWOAs) have been proposed as promising candidates for leaching mine tailings (Astuti et al., 2016; Burckhard et al., 1995; Crane & Sapsford, 2018; Li et al., 2025). They are naturally exuded from plant roots, as part of their strategy to mobilise soil nutrients, have a relatively low cost and low environmental impact (biodegradability), and have been shown to be efficient at leaching metals from mine tailings, including Cu (Crane & Sapsford, 2018; Crane et al., 2025; de Andrade et al., 2022; Jenkin et al., 2024). Citric acid, in particular, has gathered increasing attention for this purpose, especially for Cu extraction. It is a weak acid that partially dissociates in water, generating citrate, which is readily biodegradable (Crane & Sapsford, 2018; Freitas & Nascimento, 2017; Freitas et al., 2013; Jenkin et al., 2024; Renella et al., 2004).

Despite their recognised efficacy as leaching agents for mine tailings, the potential phytotoxic effects of organic acids are not often explored. This stems, in part, from the fact that they are naturally produced by plants and microorganisms, which leads to the common assumption that they are inherently non-toxic. However, the exogenous application of LMWOAs at the high concentrations required for leaching will interact with their surrounding matrix, mobilising elements that are potentially toxic to biota (e.g. Cu, Al, Pb, Cr, V). This is especially the case in mine tailings, where metal concentrations can be high and metal mobilisation can be extremely detrimental to plant and microbial survival (De Oliveira et al., 2024; Lazaro et al., 2024). For comparison, natural concentrations of organic acids in the rhizosphere are often around 0.05 mM although they may reach 8.5 mM in some cases (Sandnes et al., 2005). Thus, before these acids are employed for in situ metal recovery and for ecosystem rehabilitation on tailings, the direct effects of their application need to be better understood.

The potential of different organic acids (citric, malic, maleic, malonic, and lactic acid) for extracting Cu from legacy porphyry Cu-tailings were investigated and their potential to cause toxicity to plants growing on acid-leached tailings was explored. Different dilutions of these solvents were used to mimic a range of concentrations that are likely to occur due to their biodegradation and/or dilution in the field. Concentration is likely to influence both nutrient release and plant development, as observed by De Oliveira et al. (2024) for deep eutectic solvents. Hypotheses were that: i) organic acids (and citric acid, in particular) can mobilise high Cu concentrations in tailings; ii) associated metal mobilisation by organic acids can be detrimental to plant growth, especially at low dilution; and iii) diluted acids may still mobilise adequate levels of metals and plant nutrients in tailings, to support plant development.

## Materials and methods

### Tailings characterisation

Tailings were obtained from Tailings Storage Facility 1 (TSF1) at Philex’s Padcal Mine (Benguet, Philippines). After sieving to remove the > 2 mm fraction, metal concentrations were assessed by digestion with 50% HNO_3_ (2 h, DigiPrep block digestion, by The James Hutton Institute). Extracts were filtered and determined by inductively coupled plasma optical emission spectroscopy (ICP-OES, using a Perkin Elmer Avio 500); quality control and standard solutions were prepared from certified stocks (NIST). Total Carbon (C) and total nitrogen (N) analyses were performed at the James Hutton Institute (Aberdeen AB15 8QH), using the Dumas combustion method (FlashEA 1112 elemental analyser). Concentrations of several elements in the tailings (mg kg^−1^, or %) are presented in Table 1. Other physicochemical properties (e.g. pH, CEC, bulk density) are presented in the supplementary file (Table S1).

**Table 1:** Metal concentrations and CN analysis in tailings obtained from TSF1 at the Padcal mine (Philippines). Values are averages (n = 3) ± standard error. Average values generally reported in US soils are shown to illustrate the magnitude of tailings concentrations

Element	Concentration (mg kg^−1^)	Detection limit (mg kg^−1^)	Average US soils^1^ (mg kg^−1^)
Copper, Cu	1,440 ± 56	0.02	17
Aluminium, Al	18,565 ± 903	0.20	47,000
Calcium, Ca	4,676 ± 231	0.45	9,200
Iron, Fe	17,192 ± 823	0.10	18,000
Potassium, K	5,551 ± 187	0.50	15,000
Magnesium, Mg	18,292 ± 167	0.10	4,400
Manganese, Mn	355 ± 8	0.01	330
Phosphorus, P	633 ± 17	0.10	260
Zinc, Zn	27 ± 1	0.03	48
Vanadium, V	286 ± 15	1.0	58

CN analysis	Concentration (%)	Detection limit (%)	Global concentration in mineral soils
Carbon, C	0.04 ± 0.005	0.02	Range: 1–50% Mean: 5% (cultivated soils)^2^
Nitrogen, N	< 0.03	0.03	Range: 0.02–0.5% Mean: 0.15% (cultivated soils)^3^

^1^Shacklette and Boerngen (1984); ^2^BSSS, (2021); ^3^Weil and Brady, (2016)

### Solvent application

Stock solutions of six different organic acids (reagent grade, > 99%) were prepared with deionised water: citric acid (1 M), malic acid (DL-malic acid, 1 M), maleic acid (1 M), malonic acid (1 M), lactic acid (DL-lactic acid, 1 M), and a solution of citric + malic acid (0.5 M each). These solvents were selected based on previous studies which have demonstrated effective yet variable Cu leaching potential and phytotoxicity (De Oliveira et al., 2024; Jenkin et al., 2024; Lazaro et al., 2024). Pilot tests showed promising efficiency of malic acid in Cu removal, but high phytotoxic potential, for this reason, the solution with citric acid (citric + malic acid, 0.5 M each) was included as treatment, aiming to maintain Cu extraction while minimising toxicity.

Plastic pots containing 225 cm^3^ of fresh < 2 mm sieved tailings (~ 371 g), received 25 mL of the solvent solutions at different dilutions: 1/4, 1/16 and 1/128 (solvent/water, v/v). For each pot, these dilutions are equivalent to 250 mM (1/4), 62 mM (1/16) and 8 mM (1/128), the latter being more realistic in terms of natural concentrations in the rhizosphere (Sandnes et al., 2005). Deionised water (25 mL) was applied as a control. All treatments had four replicates. The solvents were allowed to react with the tailings for 12 days at room temperature, before being flushed with deionised water (50 mL). This period was based on previous observations in our group suggesting maximum Cu extraction by citric acid within this timeframe (data not shown). Collected leachate was diluted in 2% HNO_3_ and analysed for metals via inductively coupled plasma mass spectrometry (ICP-MS, iCAP Q Single quadrupole, Thermo Scientific, with a Cetac 560 autosampler and a nickel cone).

### Plant growth and harvest

*Brassica napus* L. seeds (forage rape) were germinated in unleached tailings and grown for six weeks before being transplanted to the acid-leached tailings. This species was selected due to its potential to tolerate and accumulate metals from contaminated soils, as is often the case in the *Brassica* genus (Mourato et al., 2015; Rossi et al., 2002; Zunaidi et al., 2024). One seedling was transplanted per pot after 12 days of acid reaction and subsequent water flushing.

Plants were grown in controlled rooms (20 °C, 65–70% humidity, photoperiod of 11 h), and watered as necessary (every two days) with deionised water. Prior to harvest, leaf chlorophyll contents (SPAD-units) were estimated by a chlorophyll meter (Minolta SPAD 502 Plus). Green cover (in cm^2^) was also determined via the Canapeo App (www.canopeoapp.com), using photos taken vertically from above each pot, at the same height for all plants (De Oliveira et al., 2024). Plants were harvested after 14 days. Roots were washed under running water and plants were split into shoots and roots before measuring fresh weight (FW). Plants were then dried in an oven at 60 °C for five days and dry weight (DW) was recorded for roots and shoots separately, so that root/shoot biomass ratios could be calculated.

### Data analyses

Data were transformed by log(x + 1) to attain normality, before analysis via Two-Way PERMANOVA (9999 permutations; *p* < 0.05) tests, followed up by pairwise comparisons (Anderson, 2017) using the software PRIMER-e v7 with PERMANOVA + add-on. For metal leaching, due to a range in values observed across dilutions, statistical tests were applied separately to each acid dilution, in which the control (water) condition was directly compared to the highest dilution of 1/128.

To investigate overall differences between the control and diluted acid (1/128) treatments, a Principal Component Analysis (PCA) was carried out with nine variables (green cover, total DW, root/shoot ratio, chlorophyll content and leachate concentrations of Al, Ca, Cu, K and Mn), to maximise the total variability explained by the analysis. Prior to the PCA, variables were transformed and z-score normalised, such that all variables were presented on the same scale (Dago et al., 2021; Legendre & Legendre, 2012). Multivariate PERMANOVA (9999 permutations, p < 0.05) was performed to determine overall differences between acids and control responses.

To visualise patterns of metal leaching potential for the different solvents at different dilutions, a metric multidimensional scaling (mMDS) was performed using Euclidean distances with nine variables (leachate concentrations of Al, Ca, Cu, Fe, K, Mg, Mn, V and Zn). Data were transformed and normalised a priori, as described previously. Multivariate PERMANOVA was performed (9999 permutations, p < 0.05) to identify different metal leaching profiles among the different solvents within each dilution. To obtain a better representation of the leaching differences between different solvent types at 1/128 dilution, an mMDS was performed using bootstrap averages (100 random samplings per group, in Euclidean space); PERMANOVA results were annotated in the generated figure.

## Results

### Leachate concentration of Cu and other elements

**Fig. 1:**
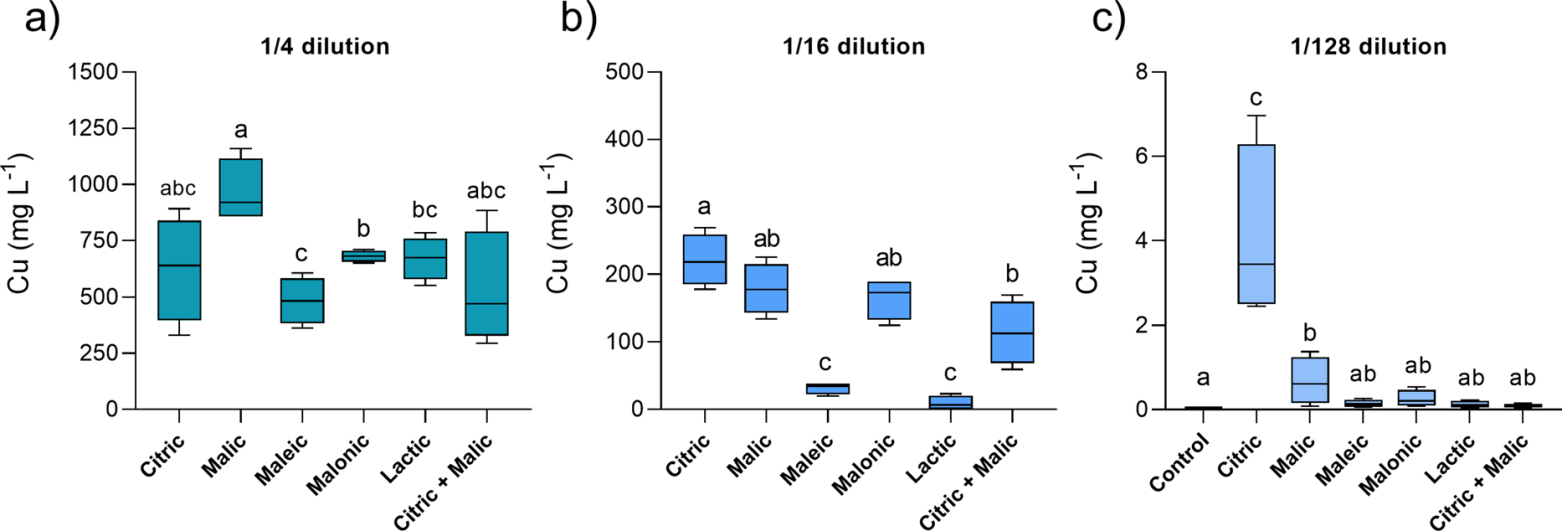
Copper concentration in leachates from acid-treated tailings after 12 days of reaction, at different dilutions: 1/4 (**a**), 1/16 (**b**) and 1/128 (**c**). Due to its low leaching capacity, control tailings (water leached), were analysed only with the 1/128 dilution treatments (c). Box plots show the minimum (lower whisker), median (line) and maximum (upper whisker) values (n = 4). Different letters represent significant differences between treatments after PERMANOVA and pairwise comparisons (*p* < 0.05). Note the scale changes between y-axes in panels (a), (b) and (c)

Acid treatment showed great potential for Cu removal after 12 days of contact with the tailings, especially at low dilutions. On average, 1/4 dilutions of citric, malic and malonic acids generated the highest mean Cu concentrations in leachate: 625, 965 and 680 mg L^−1^, respectively (Fig. 1a). These are equivalent to 11 to 17% recovery (Table S2); although citric acid results had higher variability. At 1/16 and 1/128 dilutions, citric acid had generally higher relative Cu leaching capability compared to the other acids, generating concentrations of 221 and 4.1 mg L^−1^ in leachate, respectively (Fig. 1b and c). At the highest level of dilution, only citric and malic acids leached significantly greater Cu from the tailings than the deionised water control (0.05 mg L^−1^) (Fig. 1c).

**Fig. 2:**
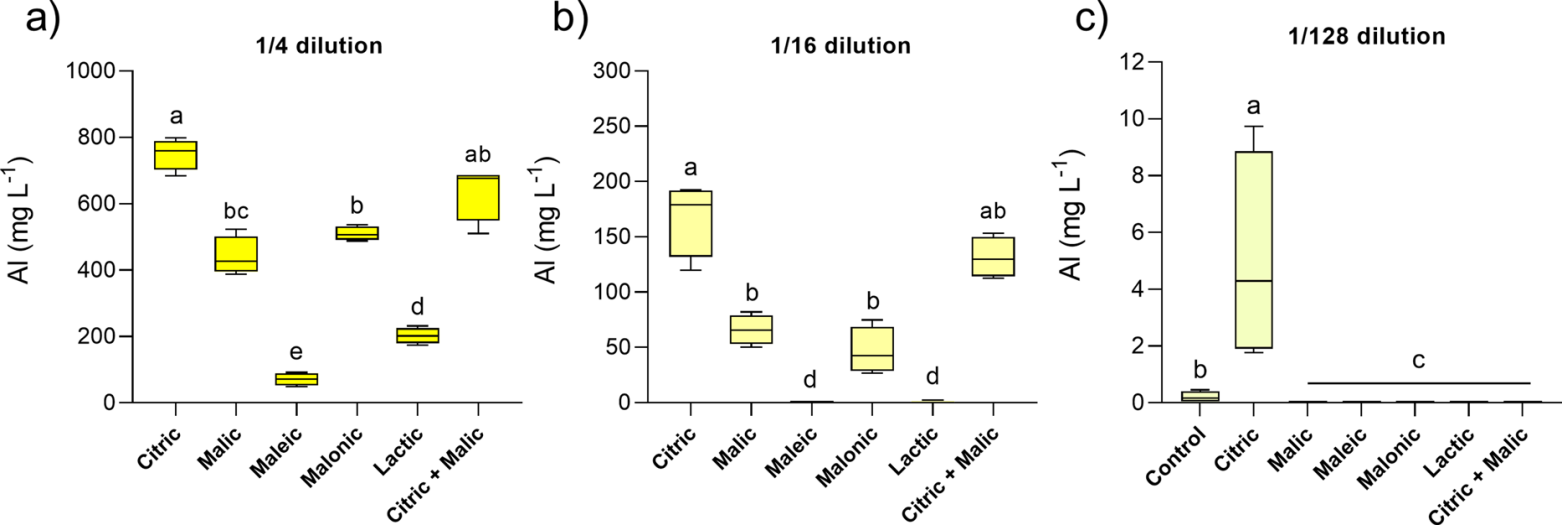
Aluminium concentration in leachates from acid-treated tailings after 12 days of reaction, at different dilutions: 1/4 (**a**), 1/16 (**b**) and 1/128 (**c**). Due to its low leaching capacity, control tailings (water leached), were compared only to the 1/128 dilution treatments (c). Box plots show the minimum (lower whisker), median (line) and maximum (upper whisker) values (n = 4). Different letters represent significant differences between treatments after PERMANOVA and pairwise comparisons (*p* < 0.05). Note the scale changes between y-axes in panels (a), (b) and (c)

Aluminium was also effectively mobilised and leached from tailings in a similar pattern to Cu, particularly at low dilutions (Fig. 2). This was especially the case in tailings treated with citric acid, with mean concentrations of 750 and 168 mg L^−1^ Al in leachate for the 1/4 and 1/16 dilutions, respectively (Fig. 2a and b). Interestingly, at the 1/128 dilution, leachates from the acid-treated tailings (< 0.01 mg L^−1^) had significantly lower Al concentrations (< 0.01 mg L^−1^) than the water control (0.2 mg L^−1^), an exception being the citric acid treatment (5 mg L^−1^) (Fig. 2c).

**Fig. 3:**
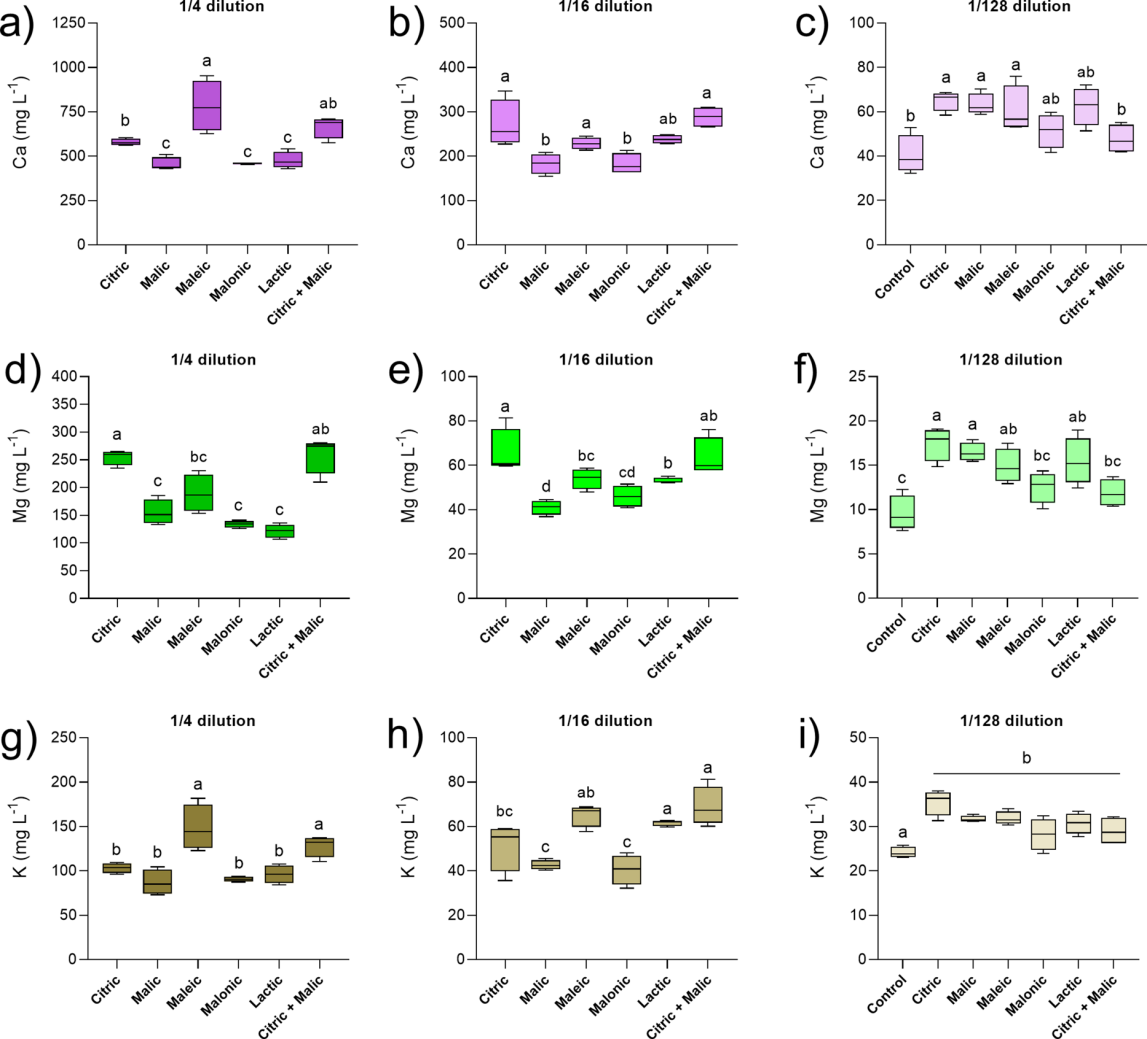
Calcium (**a**, **b**, **c**), magnesium (**d**, **e**, **f**) and potassium (**g**, **h**, **i**) concentrations in leachates from acid-treated tailings after 12 days of reaction, at different dilutions: 1/4, 1/16 and 1/128. Due to its much lower leaching capacity, control tailings (water), were compared only to the 1/128 dilution treatments (c, f, i). Box plots show the minimum (lower whisker), median (line) and maximum (upper whisker) values (n = 4). Different letters represent significant differences between treatments after PERMANOVA and pairwise comparisons (*p* < 0.05). Note the scale changes between y-axes in panels (a), (b) and (c)

Citric acid was not the most effective at leaching several other elements, including Ca and K (Fig. 3), although effects varied depending on dilution levels. At the lowest dilution of 1/4, maleic acid leachates had the highest Ca and K concentrations (Fig. 3a and g). It is important to highlight that highly diluted organic acids (1/128) were still efficient in mobilising plant macronutrients such as Ca, Mg and K in comparison to the deionised water control. While, on average, the control had 40 mg L^−1^ Ca, 10 mg L^−1^ Mg and 24 mg L^−1^ K in leachates (Fig. 3c, f, i), overall leachates from organic acids contained on average 58 mg L^−1^ Ca (i.e. 45% higher), 15 mg L^−1^ Mg (i.e. 50% higher) and 32 mg L^−1^ K (i.e. 33% higher).

### Leaching patterns at different acid dilutions

**Fig. 4:**
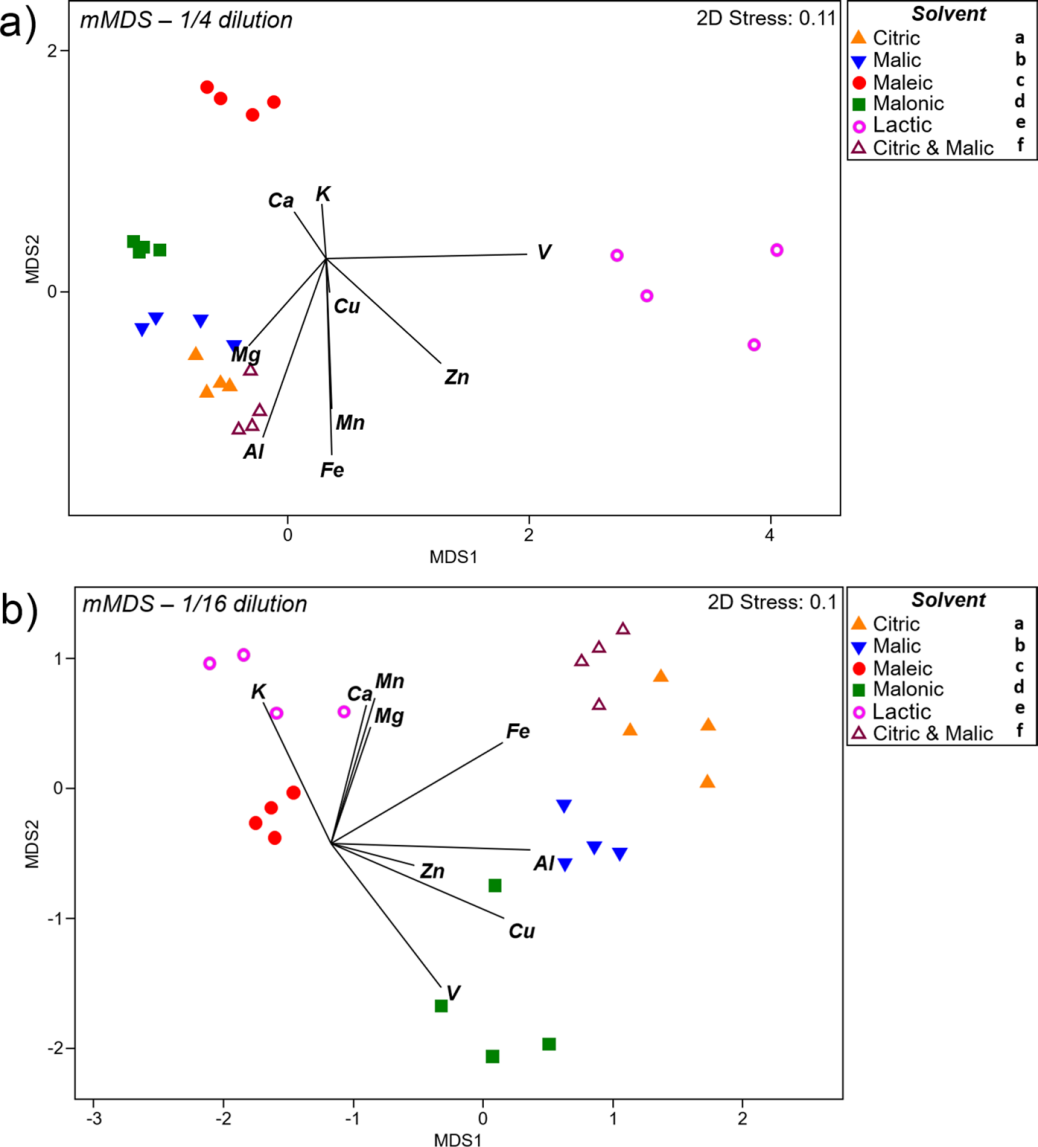
Metric multidimensional scaling (mMDS) based on Euclidean distances of nine elements leached from Cu-tailings, after application of different solvents (LMWOAs) at different dilutions: 1/4 (**a**) and 1/16 (**b**). Different letters on the legends represent significant differences between groups after PERMANOVA (9999 permutations, *p* < 0.05)

Metric multidimensional scaling (mMDS) plots were generated using leachate concentrations of nine elements (Al, Ca, Cu, Fe, K, Mg, Mn, Zn and V). These showed that leaching patterns were highly dependent on solvent dilution (Fig. S1). Therefore, mMDS was carried out for each dilution separately (Fig. 4). At the lowest acid dilution, 1/4, a clear separation is observed for lactic acid, associated with higher leaching of V compared to other solvents (Fig. 4a). Despite the short distances between citric, malic, malonic and citric + malic samples, the overall leaching pattern was significantly different among all acids for these nine elements (Fig. 4a). This was especially the case for maleic acid, which was associated with higher Ca and K leaching, but lower Al (Fig. 4a; Fig. 3a, g; Fig. 2a), while the opposite was observed for citric and malic acids. At a dilution of 1/16, all solvents presented significantly different leaching profiles (PERMANOVA, *p* < 0.05), with a particular separation for maleic and lactic acids (Fig. 4b), due to their low leaching capacity for Cu and Al (Figs. 1b, 2b).

**Fig. 5:**
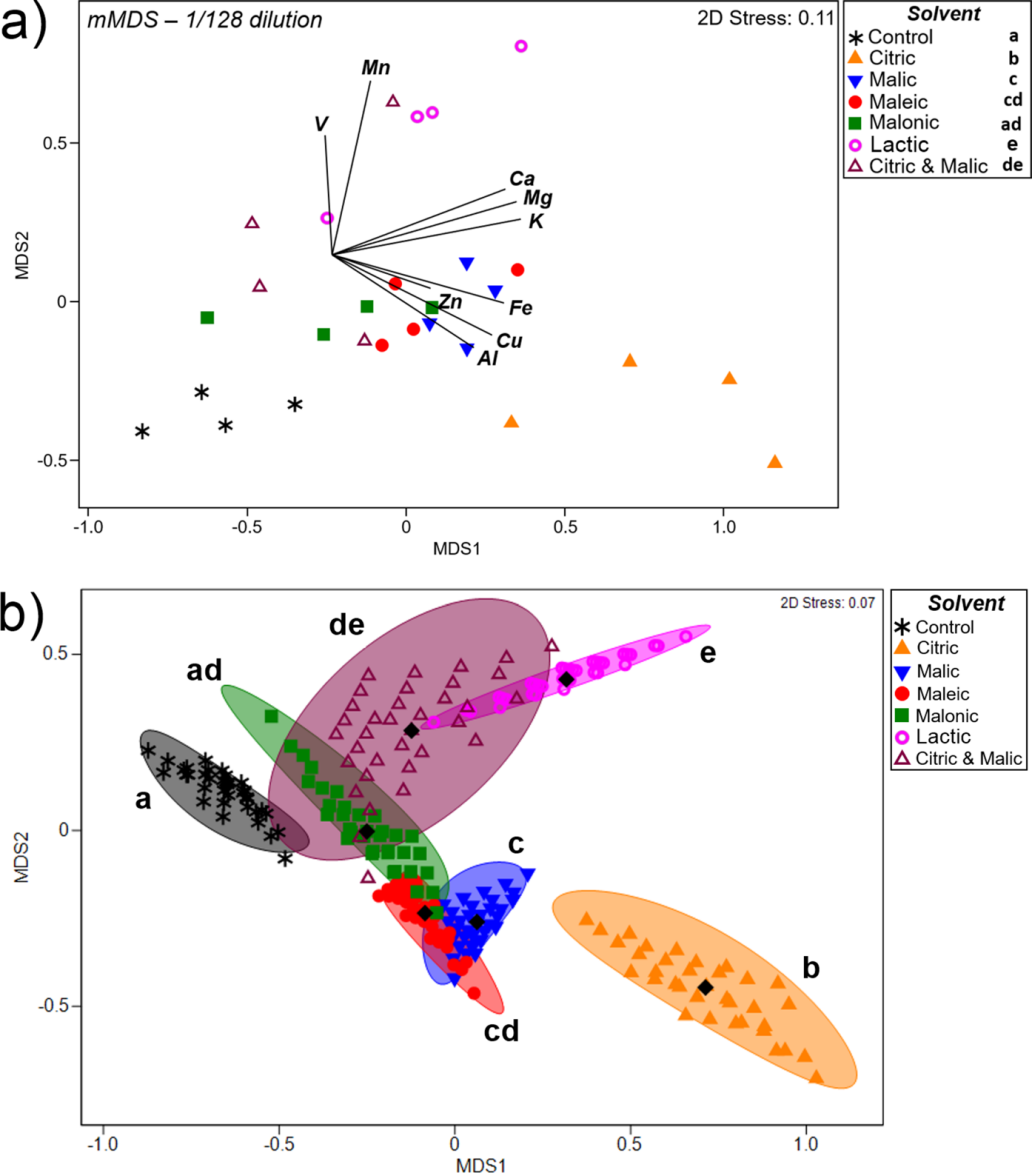
**a** Metric multidimensional scaling (mMDS) based on Euclidean distances of nine elements leached from Cu-tailings, after application of different solvents (LMWOAs) at 1/128 dilution or deionised water (control). **b** mMDS based on bootstrap averages (100 random samplings from each group) of nine elements, same as in (a); this approach simulates samplings within each group to increase n = 4 to n = 100. Different letters in the legends (a) and within the figure (b) represent significant differences between groups after PERMANOVA (9999 permutations, *p* < 0.05)

At the highest dilution (1/128), however, leaching patterns were more similar (Fig. 5a). In the control (deionised water), leaching was relatively low for all nine elements (Fig. 5a), and was statistically different from all other solvents, except malonic acid (p = 0.056). This is probably due to its low mobilisation potential for all elements, including both Mn (Fig. S2) and Mg (Fig. 3f). Citric acid was the only solvent to present a unique leaching pattern, which even differed from the citric + malic acid treatment (Fig. 5b). The unique behaviour of citric acid is reflected in its higher mobilisation of Fe (Fig. S3), Cu (Fig. 1c) and Al (Fig. 2c).

### Plant growth in acid-leached tailings

**Fig. 6:**
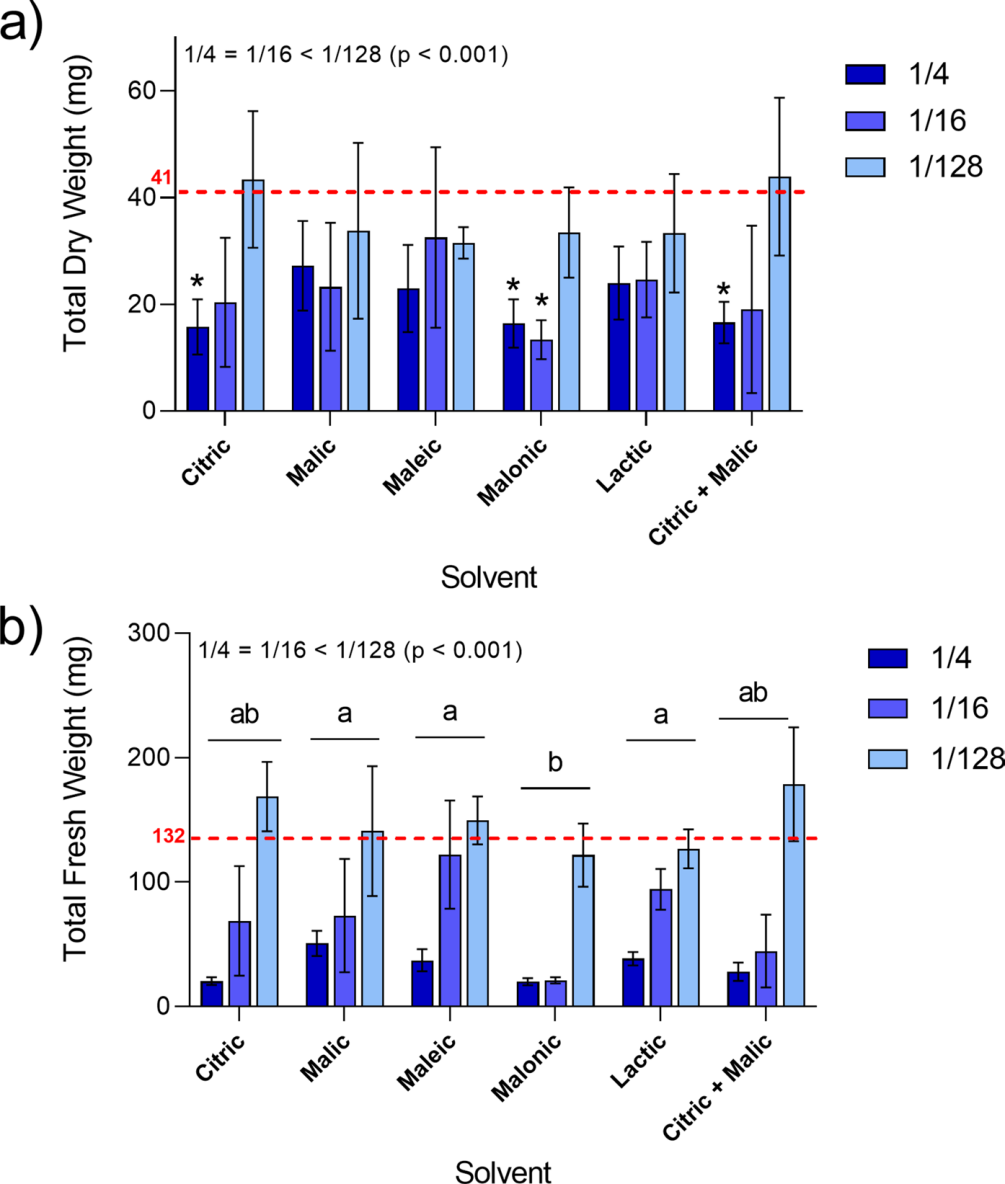
Dry (**a**) and fresh (**b**) weight of *B. napus* seedlings after 14 days growing on acid-leached tailings. Citric, malic, maleic, malonic, lactic and citric + malic acids were applied at different dilutions: at 1/4, 1/16 and 1/128 dilutions. Bars represent the means with standard errors (n = 4). Difference between dilutions is annotated on the top left (main effects) after PERMANOVA (*p* < 0.05). The red dashed line represents the average weight of control plants (deionised water) and asterisks indicate values significantly lower than controls

Overall, plants had poor development in tailings treated with the lower dilutions of organic acids (1/4 and 1/16), while little impact was observed in those treated at 1/128 dilutions (Fig. 6a). In particular, citric, malonic and citric + malic acids at 1/4 dilution were the most detrimental to plants, which had significantly lower dry weights compared to the control. A similar pattern was observed in total fresh weight, which reflects plant health and phytotoxicity at time of harvest (Fig. 6b). Malonic acid-treated tailings were specifically harmful to plants, compared to the other solvents, as the only solvent to significantly decrease dry weight at 1/16 dilution, compared to control. At 1/128 dilution, all treatments had similar fresh biomass to control plants. A breakdown of comparisons between each treatment at specific dilutions is presented in Fig. S4 (supplementary files).

**Fig. 7:**
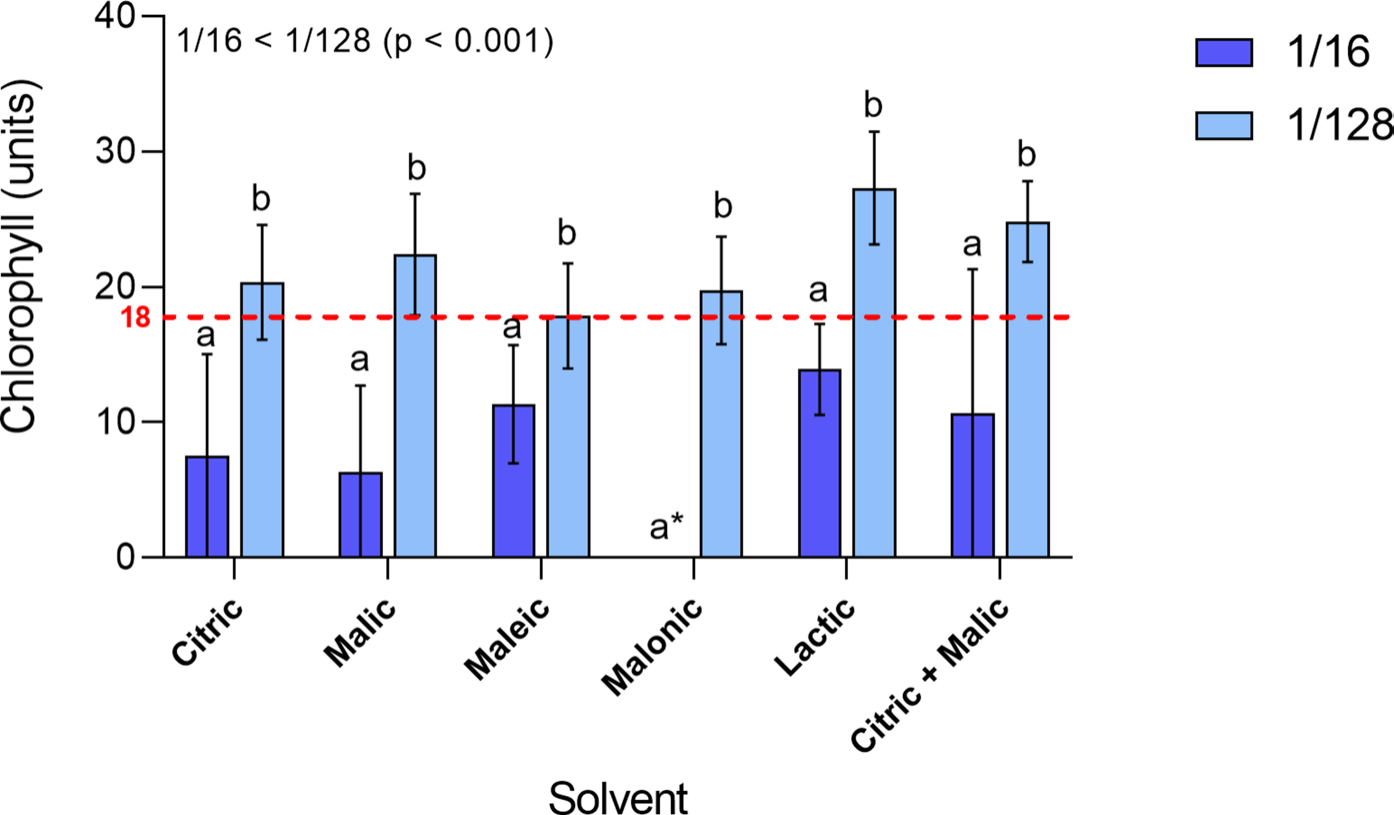
Chlorophyll content (SPAD units) in *B. napus* leaves after 14 days growing on acid-leached tailings. Citric, malic, maleic, malonic, lactic and citric + malic acids were applied at different dilutions: at 1/16 and 1/128 dilutions. Bars represent means with standard errors (n = 4). Different letters represent significant differences between treatments after PERMANOVA (*p* < 0.05). The red dashed line represents the average chlorophyll content of control plants (no solvents applied) and the asterisk denotes a plant with high necrotic rate (no chlorophyll detected)

Chlorophyll content in the leaves of plants grown on tailings treated with 1/16 and 1/128 dilutions did not differ from controls (Fig. 7, *p* > 0.05). However, a statistically higher chlorophyll content overall was observed in plants exposed to organic acids at 1/128 dilution compared to those at 1/16 dilution. Malonic acid at 1/16 dilution also caused high necrotic rates, and no chlorophyll was detected (Fig. 7). Due to pronounced necrosis in leaves in the 1/4 treatments, it was not possible to assess chlorophyll contents and, therefore, these data were excluded from this analysis.

**Fig. 8:**
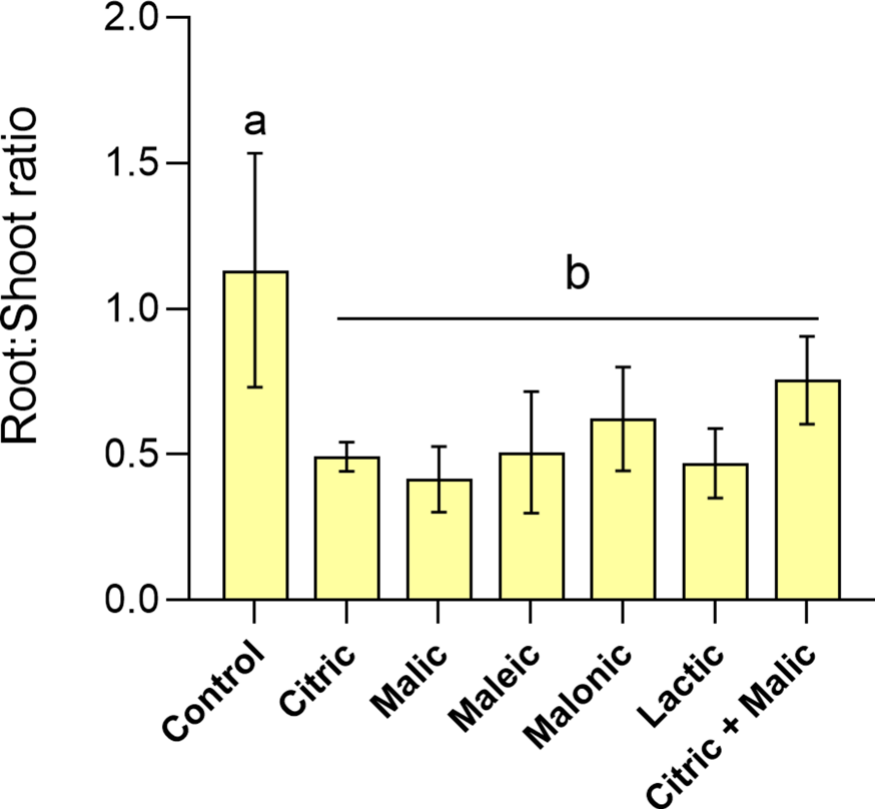
Dry root to shoot ratios of *B. napus* seedlings after 14 days growing on acid-leached tailings. Citric, malic, maleic, malonic, lactic and citric + malic acids were applied at 1/128 dilutions. Bars represent means with standard errors (n = 4). Letters represent the difference between control and acid treatments (main effects) after PERMANOVA (*p* = 0.018)

Although root dry weight did not differ between control plants and those treated by diluted organic acids (1/128) (Fig. S5c) the root/shoot ratios were significantly higher in control plants compared to those of acid-treated plants (main effect) (Fig. 8).

### Multivariate analyses of leachates and plant responses from diluted acid treatments

**Fig. 9:**
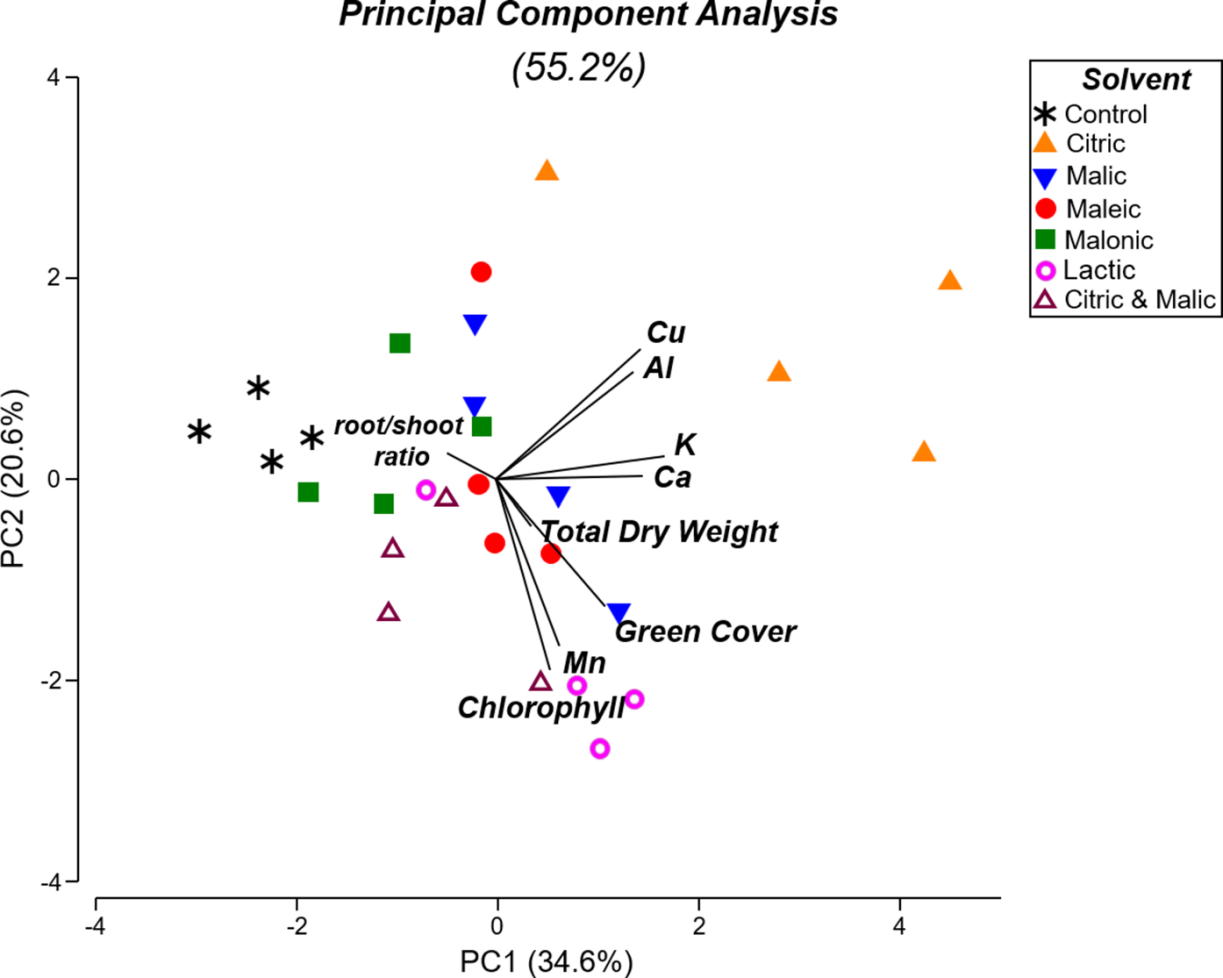
**a** Principal Component Analysis (PCA) based on Euclidean distances and nine variables, after application of different solvents (organic acids) at the highest dilution of 1/128, or deionised water (control). Variables: green cover, total dry weight, dry root/shoot ratios, chlorophyll, and leachate concentrations: Al, Ca, Cu, K, and Mn

A PCA was performed using only data from the control and the diluted acid treatments (1/128). This was able to explain 55.2% of the total variability, largely carried by the vectors for leachate element concentrations (Cu, Al, K, Ca) (Fig. 9). The plot clearly shows little variability in responses from control conditions (points closer together), but higher variation in citric acid treatments (points spread apart). Acid treatments are generally associated with high metal leaching (particularly for citric and malic acids), with chlorophyll and green cover particularly for lactic acid, whilst control conditions are mainly associated with higher root/shoot ratios. Vectors for chlorophyll, green cover and Mn concentrations are positively associated, and the same is observed between Cu and Al. The PCA also shows an inverse relationship between root/shoot ratios and total dry weight or green cover.

PERMANOVA detected significant multivariate differences between solvents (p < 0.001, Fig. 9), especially for control conditions, which differed from all other treatments, except for malonic acid (p = 0.169). Responses to citric acid were significantly different from those for all other acids (p < 0.05), except for malic acid, which showed some similarities (p = 0.055). Interestingly, the mixture of citric (0.5 M) and malic (0.5 M) acids showed similar responses to the single malic acid treatment (p = 0.146), but not to the citric acid treatment (p = 0.027). These differences are also observed in leachate concentrations of Cu (Fig. 1c) and Al (Fig. 2c).

## Discussion

### Metal leaching potential of different organic acids

Organic acids removed between 8 and 17% of Cu from tailings at 1/4 dilution (250 mM) after only 12 days of contact. These results show that these acids can effectively leach metals from raw tailings, as expected from previous work (Crane & Sapsford, 2018; Jenkin et al., 2024; Ke et al., 2020), and that the leaching potential of each acid varies depending on their molar concentration (dilution factor). This experiment was not optimised to maximise Cu extraction as carried out in other work, which reported 54% Cu removal from tailings by citric acid leaching (Jenkin et al., 2024). Here, citric and malic acids consistently showed strong Cu removal compared to the other solvents, across all dilutions (1/4; 1/16 and 1/128). Ke et al. (2020) reported an 11.1% Cu removal from contaminated soil by citric acid (0.1 M) after several applications over 35 days, which were deemed mainly exchangeable, carbonate-bound and oxide-bound Cu fractions. Although, in the current experiment, at 1/4 dilution (250 mM) malic acid promoted slightly more Cu leaching than citric acid, at higher dilutions citric acid leached more Cu. For example, at 1/128 (~ 8 mM) dilution, citric acid leached approximately 6-times more Cu than malic acid. Similar results have been reported in experiments with iron tailings, in which citric acid extracted approximately twice as much Cu as malic acid (both at 5 mM) after 20 days of reaction (Geng et al., 2020).

These tailings samples were particularly rich in Al and Mg (Table 1) and, therefore, high leaching of both elements was expected. Unlike for Cu, these elements were generally most effectively leached by either citric acid or by the citric + malic acid mixture, but not malic acid alone. Although many studies focus mostly on reporting results for valuable and/or ecotoxic target metals from contaminated substrates, such as Cu, Zn, lead (Pb) and nickel (Ni) (Buckhard et al., 1995; Ke et al., 2020; Geng et al., 2020; Lima et al., 2025), other elements such as Al and Mg are highly relevant if there are plans for ecosystem rehabilitation, given their phytotoxic and nutritional roles in plant establishment and growth (De Oliveira et al., 2024). However, it has been shown in leaching experiments targeting rare earth elements, that citric acid indeed mobilises high amounts of non-target elements such as Al (Shalchian et al., 2024). Similarly, for low-grade saprolitic ores, Astuti et al. (2016) reported that citric acid (1 M) was highly selective for Ni leaching compared to other acids (e.g. sulphuric and oxalic), but still very effective in mobilising Mg, leaching about 60% after 15 days. This is probably due to its unique chemical properties including strong chelating ability with divalent metals. Despite the leaching of non-target Al, here we show that citric acid presented high selectivity for Cu over Al. For instance, at 1/4 dilution, citric acid leached around 11% (Table S2) of the Cu pool (1,440 mg kg^−1^, Table 1), and only 13% of Al, despite the Al pool being 13 times higher than Cu (18,565 mg kg^−1^, Table 1). It is important to consider that Al-citrate complexes can be re-sorbed onto mineral particles. However, it has been shown that while citrate increases Al adsorption in soils, higher desorption rates occur at citrate concentrations above 0.5 mM (Xu et al., 2005). The concentrations used in this work (> 8 mM) were all well over this threshold.

There were clear differences in leaching profiles among the different acids (Fig. 4). To the best of our knowledge, the approach of mMDS, followed by PERMANOVA has not previously been applied to differentiate metal leaching patterns between organic acids. Here, it is evident that each acid has its own unique pattern of metal leaching from the Cu-tailings (1/4 and 1/16 dilutions), although leaching became somewhat similar when they were highly diluted (1/128) and sometimes similar to water (e.g. malonic acid). An exception was citric acid which, even at high dilutions, still generated its own unique pattern of metal mobilisation, which reflected its efficient mobilisation of Cu even in low concentration (Figs. 4c).

The distinctiveness of citric acid’s leaching profile compared to the other LMWOAs assessed here may be due to its three carboxyl groups, thus providing three metal binding sites (tridentate), while the other acids only have one or two carboxyl groups (Zabiszak et al., 2018). Having a higher number of carboxyl groups could be the reason for the higher Cu and Al leaching found for citric acid (1/128) compared to the other diluted acids, including the citric + malic acid treatment. Although not investigated in this work, it is possible that different biodegradability rates had a stronger effect on the leaching potential of LMWOAs when these were highly diluted (1/128), but not when concentrated (1/4 and 1/16). Considering that molecular structure and size influence biodegradability rates (Kim et al., 2023), it is likely that the diluted citric acid was slower to biodegrade than the other acids, allowing more contact time to react with the material and mobilise more Cu.

It is worth noting that, at the highest dilution level, most acids were able to mobilise more plant nutrients than the water-treated tailings, such as Ca, Mg and K, which may provide better nutritional conditions for plants establishing in these tailings, as suggested by De Oliveira et al. (2024).

### Plant development in acid-leached tailings

The deleterious nature of mine tailings for plant growth is well known. This can arise from their high metal contents, lack of nutrients, lack of organic matter or sub-optimal physical structure for root development (Mendez and Meier, 2007; Yuan et al., 2016; Wang et al., 2017). Thus, without any fertilisation or organic amendments (e.g. biochar, manure, compost), poor plant growth was expected in these Cu tailings. This was confirmed by the very low dry biomass observed for *B. napus,* which did not exceed 60 mg in any treatment despite eight weeks of growth. For comparison, in non-contaminated soils *B. napus* seedlings can have over 300 mg of dry weight within the first four weeks (Lacalle et al., 2018).

In these tailings, the lack of organic matter and, in particular, nitrogen (< 0.3%), are likely to be the main stressors for plant development, limiting their growth due to N deficiency and making them more vulnerable to toxicity (Ye et al., 2022). For instance, the addition of 5% of sugarcane compost into Cu and Pb–Zn tailings was shown to effectively increase N concentrations from 0.15 to 18.6 g kg^−1^ and allowed better plant survival (*Iseilema vaginiflorum*) in comparison to raw tailings (Yuan et al., 2016).

Overall, organic acid additions were detrimental to plant growth at 1/4 and 1/16 dilutions (250 mM and 62.5 mM, respectively) as expected. However, at the 1/128 dilution (~ 8 mM) plants presented similar or better growth and chlorophyll contents compared to control tailings. After 12 days of acid application, it is clear that plants under 1/4 and 1/16 dilutions experienced phytotoxicity. This was most likely the result of Cu and Al excess, causing foliar necrosis, chlorophyll degradation, stunted growth and short roots (Mir et al., 2021; Shetty et al., 2021). Toxicity was particularly apparent in malonic acid treatments, which did not mobilise as much Cu and Al as citric and malic acids, but also mobilised less of the nutrient elements (e.g. Ca, Mg and K) than the other acids. These elements could have counteracted Cu and Al uptake and toxicity (Chen et al., 2013, 2019; Juang et al., 2021).

Although *B. napus* is known to accumulate high concentrations of metals, such as Cd, Cu and Zn, the extremely high availability of Cu after acid treatment was probably limiting to seedling growth. Cu is well known to exert high toxicity at these levels, which may have been exacerbated by the other mobilised metals (Soltangheisi et al., 2024). Plants require low amounts of Cu for optimal growth (Cakmak et al., 2023) and concentrations above 10.5 µM Cu (0.6 mg L^−1^) can be enough to cause phytotoxicity (Cook et al., 1998), although this will depend on the plant species and exposure time. It has been demonstrated under hydroponic conditions that *B. napus* seedlings exposed continuously to 50 µM Cu (3.17 mg L^−1^) for 14 days had an 80% decrease in biomass compared to control conditions (Feigl et al., 2015). This level of Cu exposure is much lower than the Cu concentrations measured in the leachates collected in this experiment, which were over 250 mg L^−1^ in 1/4 dilutions and over 100 mg L^−1^ in 1/16 dilutions. In other plant species, such as *Hordeum vulgare*, the toxicity threshold may as low as 2.5 mg L^−1^ Cu (Zhang et al., 2013).

From a recovery and remediation perspective, more concentrated citric acid showed great potential for leaching Cu from the tailings. However, high concurrent availability of non-target metals, such as Al and Mn, also occurred. Al, in particular, is known for its phytotoxic effects, especially in highly weathered and acidic soils (Shetty et al., 2021). Moreover, these tailings possess little to no organic matter (Table 1) that could provide effective adsorption sites for the released Al ions, further enhancing their availability in the rhizosphere (Li et al., 2022). In hydroponics, a concentration of 2.7 mg L^−1^ (100 µM) Al was shown to cause deleterious effects in roots of *B. napus*. This concentration was much lower than that observed in the concentrated acid leachates collected from the tailings in this study.

Lima et al. (2025) have shown that the application of citric acid (20 mM) in ultramafic soils resulted in high Ni mobilisation, but this was associated with a decrease in biomass (30 to 44% lower) in two Ni hyperaccumulator plants. Lima et al. also observed concurrent mobilisation of many other metals, including Mn(II) and Co(II). Of course, these effects are highly dependent on plant species, metal concentrations in the substrate and other variables such as time of exposure and acid concentration. For instance, *Sedum alfredii* grown in citric acid-treated (10 mM) soils contaminated by Pb, Zn and Cd, exhibited only a slight decrease in photosynthetic efficiency compared to un-treated controls (Li et al., 2021).

In addition to their ability to mobilise toxic elements such as metals, high concentrations of organic acids can be inherently phytotoxic (Lee et al., 2006). However, in this experiment, acids were flushed after 12 days of contact with the tailings, and frequent watering and natural degradation are likely to have decreased residual concentrations even further during plant growth. It is unlikely, therefore, that the toxicity observed here is due to the acids themselves (Agnello et al., 2015). Instead, more plausibly, phytotoxicity resulted from an increase in metal mobility. It is important to consider that despite these solvents being more biodegradable and of lower strength than most mineral acids, their phytotoxicity potential will ultimately depend on their interaction with the substrate matrix (De Oliveira et al., 2024).

### Plant development under diluted acid applications

Despite the toxicity caused by the concentrated acids, when diluted, these solvents can improve plant growth and tolerance, as well as foliar metal accumulation. In *B. napus,* Menhas et al. (2024) showed that application of 2.5 mM citric acid in a Cd-contaminated soil was able to improve plant physiological responses, despite increasing Cd uptake; these included higher activity of antioxidant enzymes, lower lipid peroxidation and up-regulation of genes involved in Cd transport and detoxification. Similar observations were reported by De Oliveira et al. (2024), after applying diluted deep-eutectic solvents (8 mM) in Cu-tailings, which led to enhanced biomass, root area and chlorophyll contents of *Plantago lanceolata* seedlings. Similarly here, it is clear that organic acids at high dilutions promoted higher nutrient mobilisation than a water control. Whilst these were not sufficient to significantly enhance *B. napus* biomass or chlorophyll, they did decrease root/shoot ratios. Higher biomass allocation towards roots instead of shoots is a common response to nutrient deficiency in the environment, as plants invest in root growth to better scavenge for water and nutrients (Lynch et al., 2012). The PCA suggests plant in the water control invest more in roots instead of shoots, while organic acid-treated plants at high dilution (1/128) exhibited higher chlorophyll, green cover and total biomass, possibly due to higher nutrient availability (e.g. Ca, K), allowing more investment in shoot growth. It is important to mention that root dry weights in the control and diluted acid treatments were not significantly different; only their root/shoot ratios, which suggests that this difference in ratios is mainly driven by nutrient availability rather than metal toxicity.

One of the reasons for testing diluted acids was to simulate the natural biodegradation, dilution and dispersion of these solvents. LMWOAs can be biodegraded within hours in soils (van Hees et al., 2005). Citric acid for instance, was shown to degrade within 5 to 10 days after application in contaminated soils (Freitas & Nascimento, 2017; Freitas et al., 2013), while in a limed forest soil, van Hees et al. (2003) reported that the half-life of citrate (0.5 mM) in surface horizons was less than 6 h. Less information is available for biodegradation rates of organic acids in mine tailings. Rates are likely to be lower than in soils, due to lower microbial activity and (probable) microbial diversity. In these same legacy Cu-tailings, Lazaro et al. (2024) found extremely low abundance of microbial DNA, low biomass and an impoverished microbial community, which would be expected to slow organic acid biodegradation. Nevertheless, some biodegradation is likely to occur which could decrease concentrations over a few days. The biodegradability of organic acids in tailings still needs to be better understood and additional work is needed to establish rates and controls.

## Conclusions

The Cu extraction potential of different organic acids from legacy Cu-tailings over a range of dilutions were evaluated, as well as their impact on subsequent plant development. Citric acid and malic acids had the highest Cu leaching capacity (generating leachate concentrations of 625 mg L^−1^ and 965 mg L^−1^, respectively). These acids effectively mobilised non-target metals, with a clear inverse relationship between acid concentration and leaching efficiency. At 1/128 dilution (~ 8 mM), citric acid stimulated the highest mobilisation of several plant nutrient elements (e.g. Cu, Mg, Ca, K), as well as Al, although at non-toxic levels. Our leachate analyses show that application of different LMWOAs result in unique element mobilisation profiles, which deserve further exploration to better understand the mechanisms behind these patterns.

When concentrated (1/4; 250 mM), application of organic acids to Cu-tailings severely hindered the growth of *B. napus*, most likely due to the mobilisation of both Cu and Al. However, at high dilution (1/128; ~ 8 mM), plant biomass and chlorophyll contents were comparable to those grown in the water control, but with significantly lower root/shoot ratios, suggesting better nutritional status in the acid-treatments than in the control.

Due to its widespread accessibility and relatively low cost, together with the comparable (or better) results from citric acid in relation to the other organic acids evaluated here; we highlight citric acid as the most promising solvent for remediation of Cu-tailings. It can initially extract high amounts of Cu and Al (and Mn and Fe to some extent) and, following biodegradation and dilution by rainfall, it may promote plant growth by mobilising plant nutrients at lower and beneficial concentrations. Nevertheless, its leaching efficiency and biodegradation rates should be further investigated in order to maximise Cu removal and subsequent plant development directly on tailings. Moreover, to properly allow successful revegetation, organic matter and nitrogen inputs will still be necessary to improve microbial activity, substrate aggregation and plant nutrition.

## Electronic supplementary material

Below is the link to the electronic supplementary material.

Supplementary Material 1

## Data Availability

Data will be made available upon request.
