# Supplementary material for: Copper extraction and phytotoxicity of organic acid leached mine tailings in Brassica napus
Source: Environ Geochem Health. 2026 Apr 14;48(7):299. doi: 10.1007/s10653-026-03188-7 (PMC13079549; doi:10.1007/s10653-026-03188-7)
Supplement: Supplementary file 1 — Supplementary Material 1 [file 10653_2026_3188_MOESM1_ESM.docx]

**Supplementary Files**

**Title:** Copper extraction and phytotoxicity potential of organic acid leached mine tailings

Vinicius H. **De Oliveira**, Sarah **Duddigan**, James **Symons**, Michael J. **Whelan**, Vimalnath **Selvaraj**, Andrew P. **Abbott**, Rich Crane, Gawen R. T. **Jenkin**, Mark **Tibbett**

| 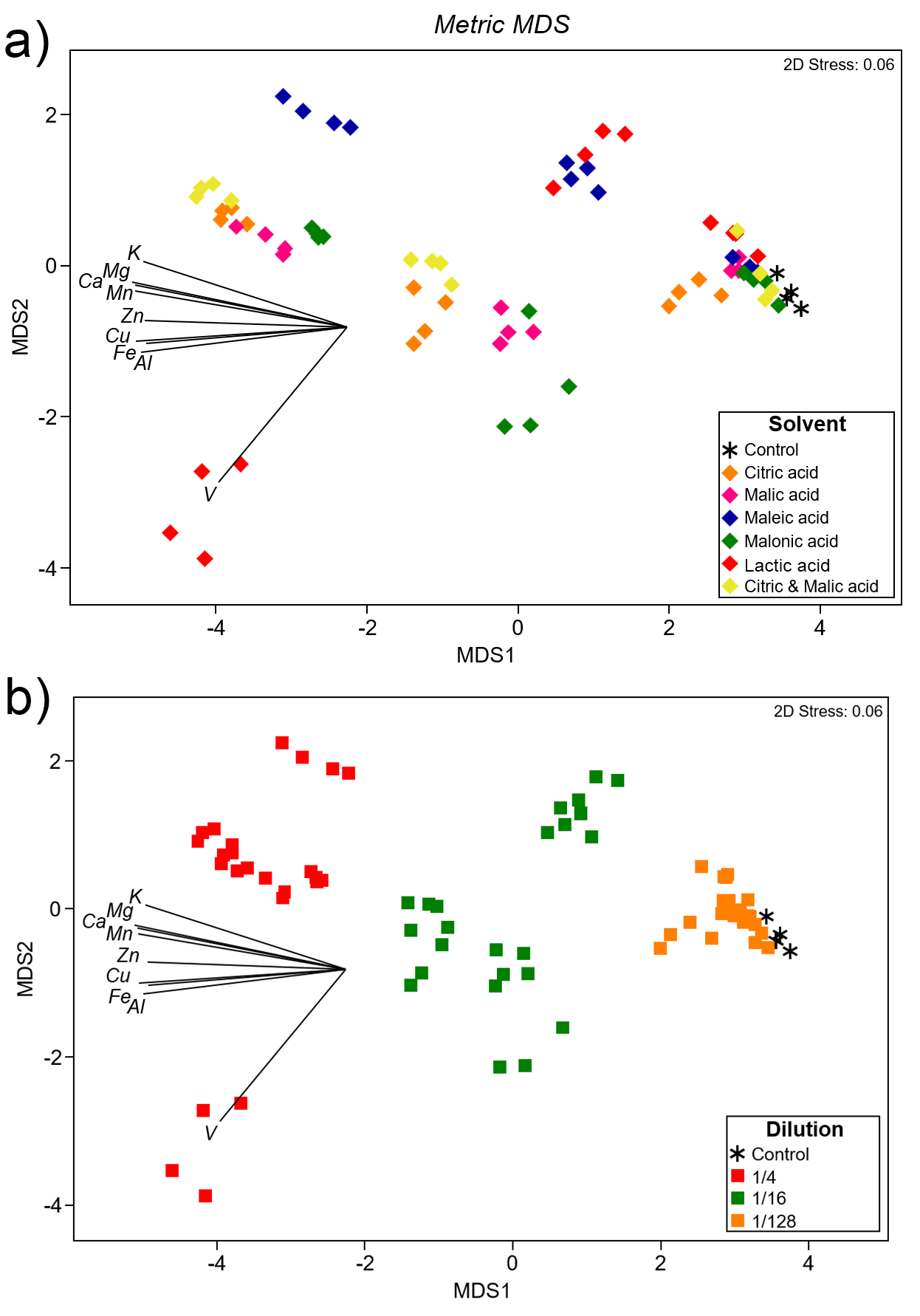 |
| --- |
| **Figure S1** – Metric multidimensional scaling (mMDS) based on Euclidean distances of 9 elements leached from Cu-tailings, after application of different solvents (organic acids or water as control) at three different dilutions: 1/4, 1/16 and 1/128. The ordinations are the same in both figures, but top figure shows the different solvents (a), and bottom figure shows their distribution according to dilutions (b). |

| 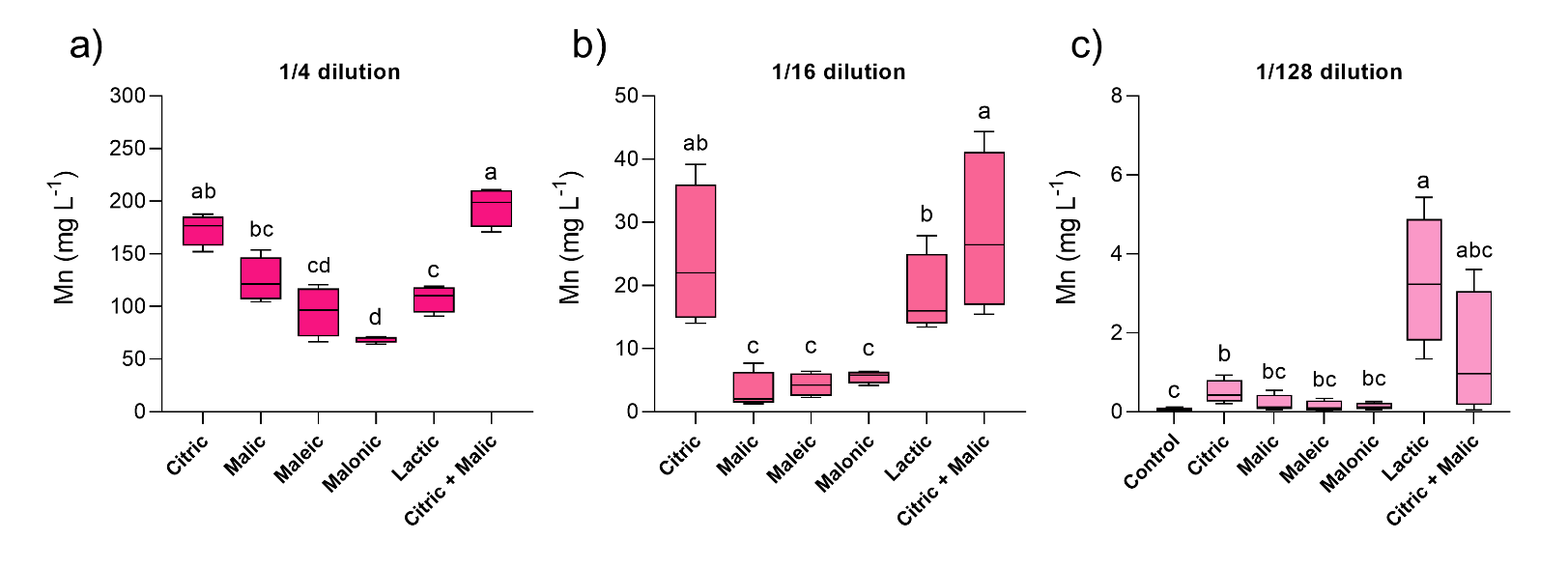 |
| --- |
| **Figure S2** – Manganese concentration in leachates from acid treated tailings after 12 days of reaction, at different dilutions: 1/4 (a), 1/16 (b) and 1/128 (c). Due to its much lower leaching capacity, control tailings (water), were compared only to the 1/128 dilution treatments (c). Box plots show the minimum (lower whisker), median (line) and maximum (upper whisker) values (n=4). Different letters represent significant differences between treatments after PERMANOVA and pairwise comparisons (p < 0.05). Please note the scale changes between Y axes in figures a), b) and c). |

| 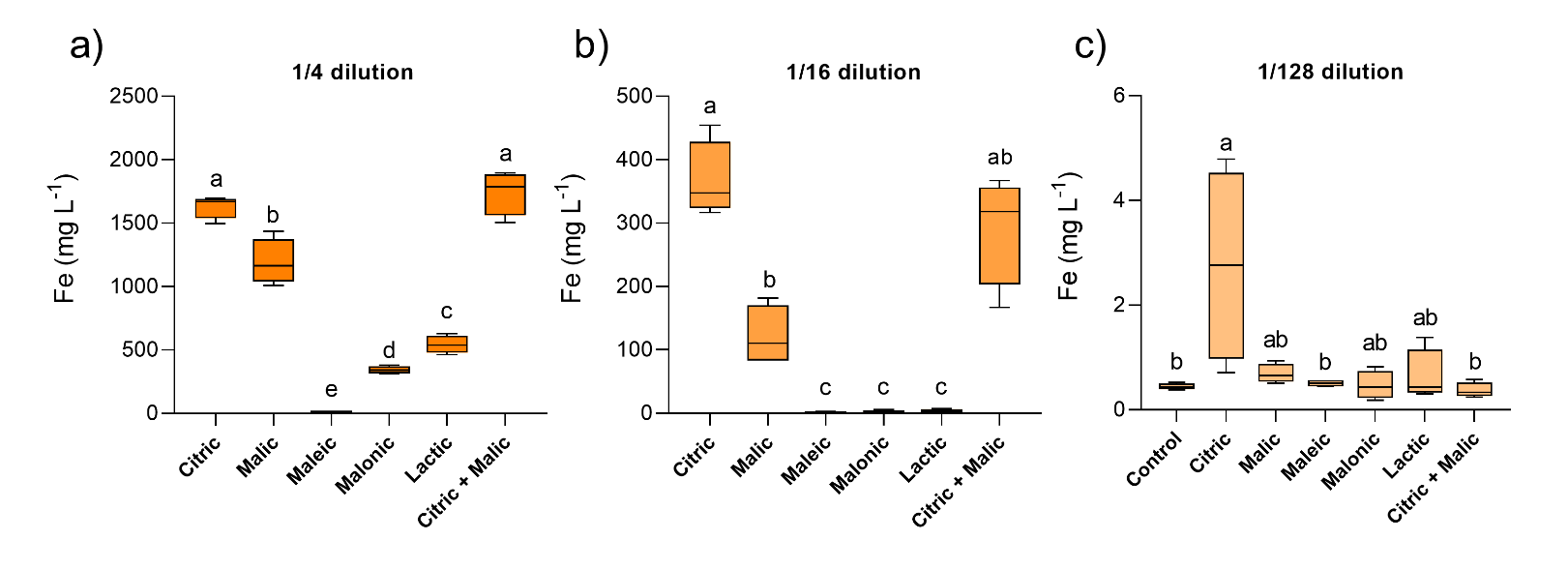 |
| --- |
| **Figure S3** – Iron concentration in leachates from acid treated tailings after 12 days of reaction, at different dilutions: 1/4 (a), 1/16 (b) and 1/128 (c). Due to its much lower leaching capacity, control tailings (water), were compared only to the 1/128 dilution treatments (c). Box plots show the minimum (lower whisker), median (line) and maximum (upper whisker) values (n=4). Different letters represent significant differences between treatments after PERMANOVA and pairwise comparisons (p < 0.05). Please note the scale changes between Y axes in figures a), b) and c). |

| 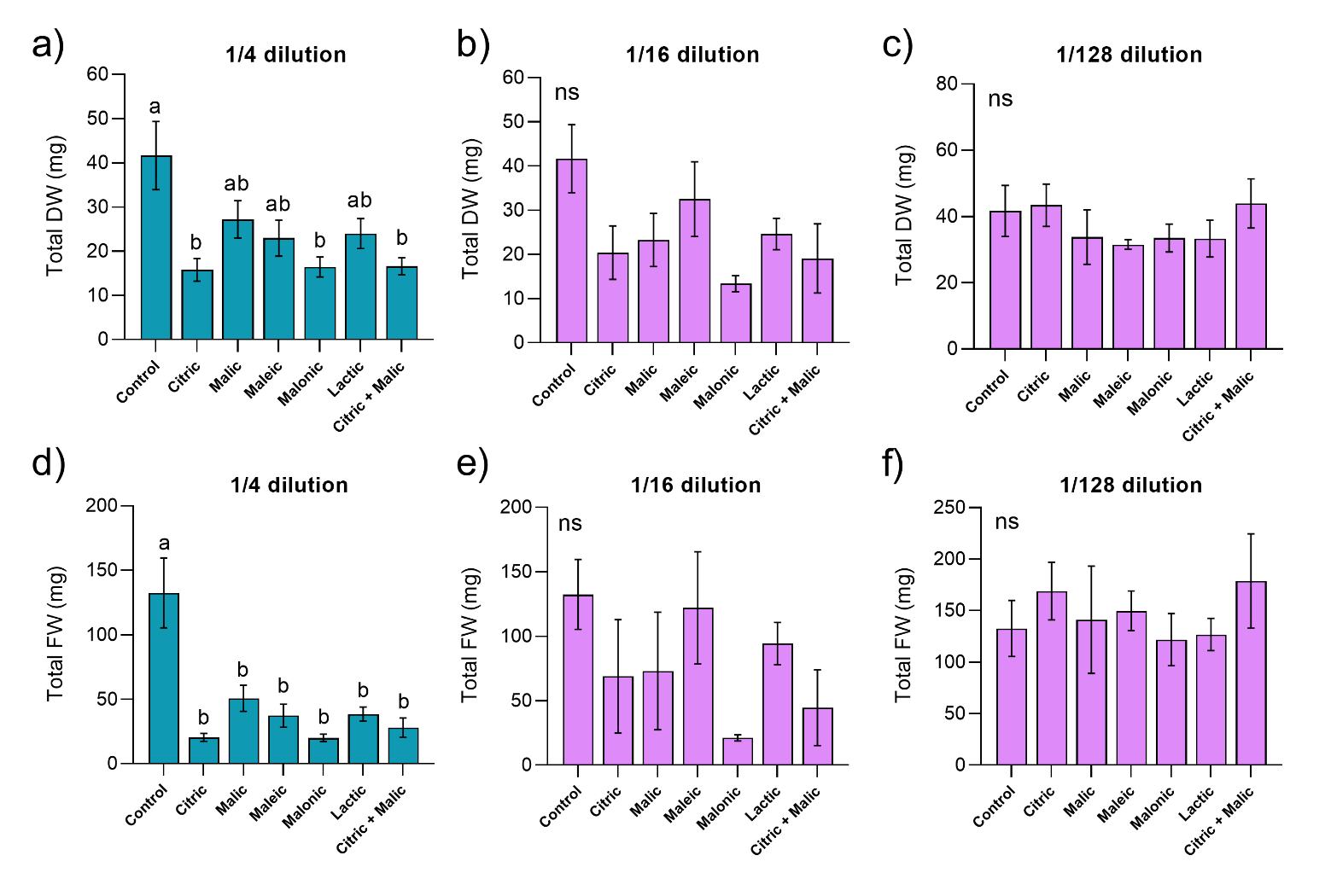 |
| --- |
| **Figure S4** – Total dry (a, b, c) and fresh (d, e, f) weights of *B. napus* seedlings after 14 days growing on acid-leached tailings. Citric, malic, maleic, malonic, DL-lactic and citric + malic acids were applied at different dilutions: at 1/4, 1/16 and 1/128 dilutions. Bars represent the means with standard errors (n=4), and letters represent significant pairwise differences after PERMANOVA (p < 0.05). n.s.: not significant. |

| 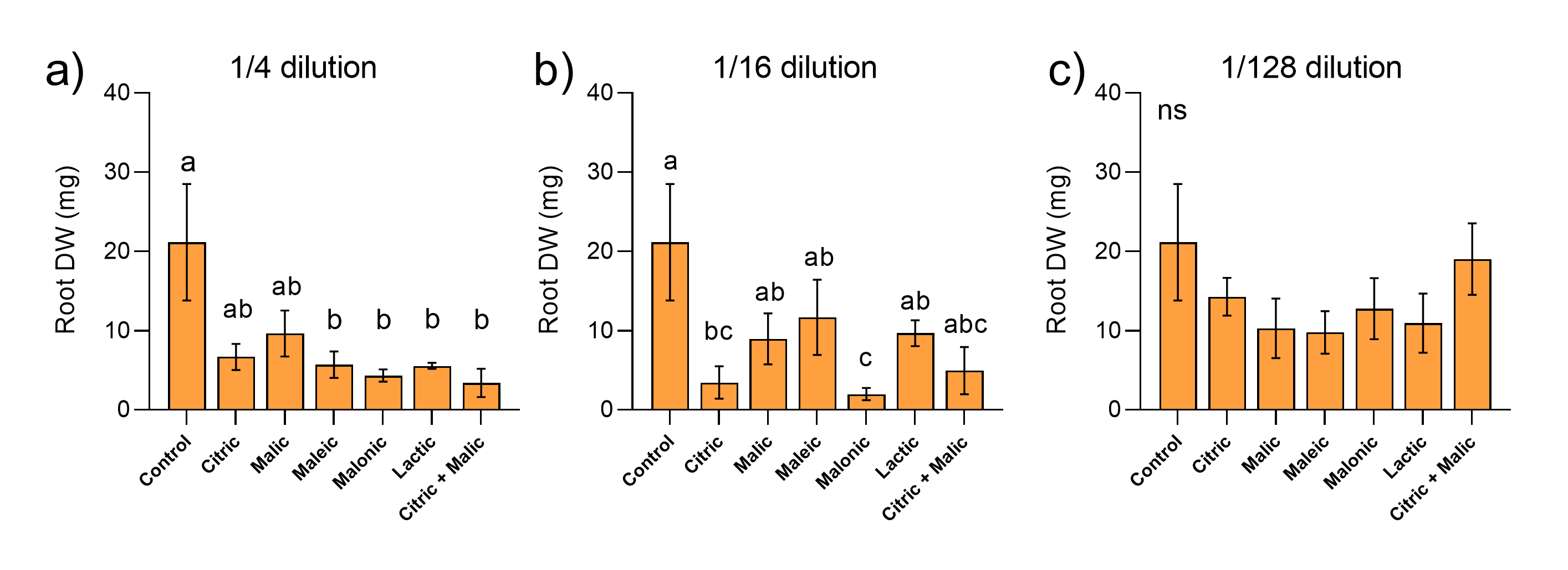 |
| --- |
| **Figure S5** – Root dry weights of *B. napus* seedlings after 14 days growing on acid-leached tailings. Citric, malic, maleic, malonic, DL-lactic and citric + malic acids were applied at different dilutions: at 1/4 (a), 1/16 (b) and 1/128 (c) dilutions. Bars represent the means with standard errors (n=4), and letters represent significant pairwise differences after PERMANOVA (p < 0.05). n.s.: not significant. |

| 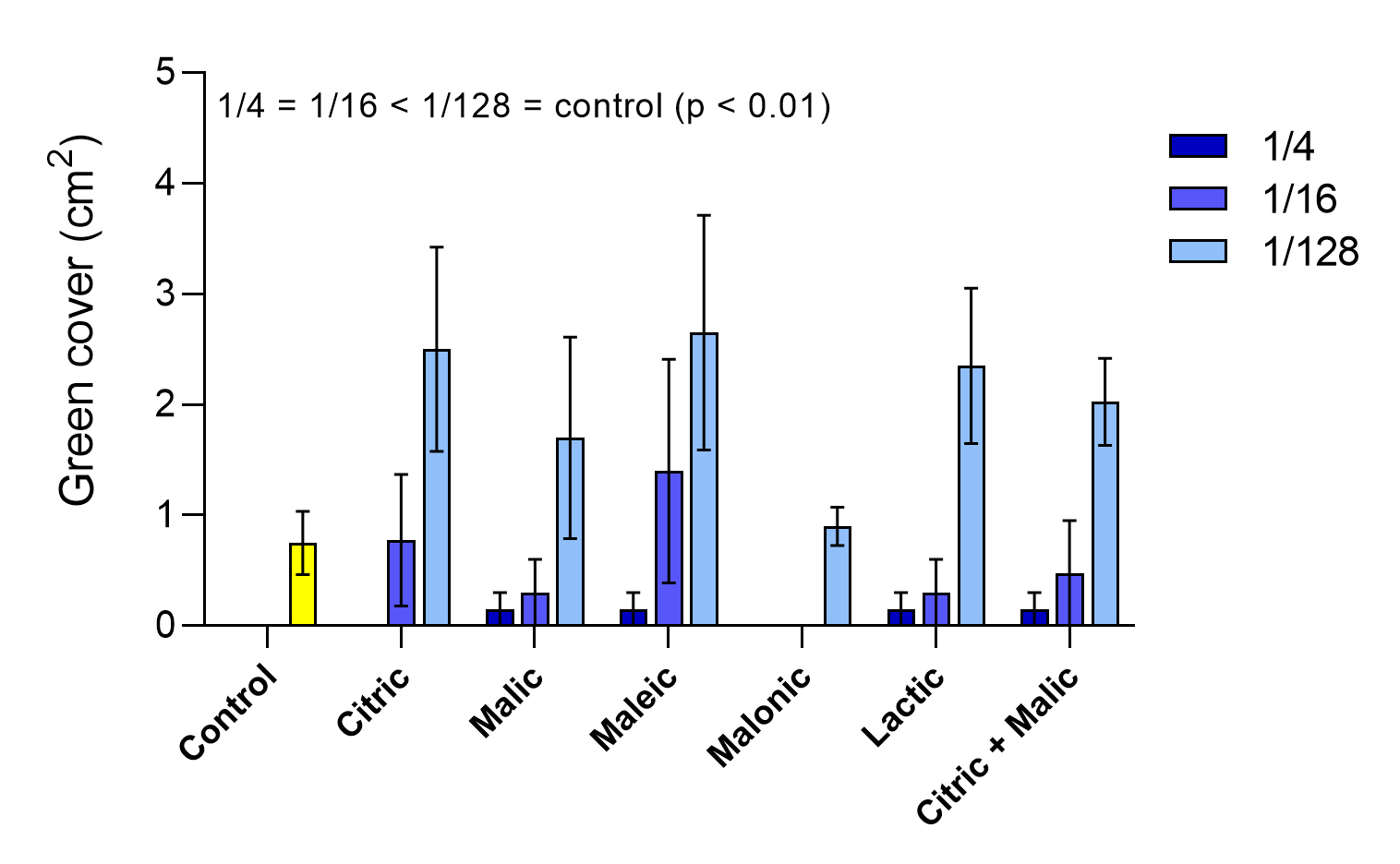 |
| --- |
| **Figure S6**. Green cover (cm^2^) of *B. napus* seedlings after 14 days growing on acid-leached tailings. Citric, malic, maleic, malonic, DL-lactic and citric + malic acids were applied at different dilutions: at 1/4, 1/16 and 1/128 dilutions. Bars represent the means with standard errors (n=4). Difference between dilutions is annotated on the top left (main effects) after PERMANOVA (p < 0.05). |

| **Table S1** – Physicochemical characteristics of untreated tailings obtained from Tailings Storage Facility 1 (TSF1) at Philex’s Padcal Mine (Benguet, Philippines)^1^. | | |
| --- | --- | --- |
| **Parameter** | **Value** | **Units** |
| Sand | 71 | % |
| Silt | 26 | % |
| Clay | 4 | % |
| Textural classification | Loamy sand | - |
| Cation Exchange capacity (CEC) | 3.4 | meq 100 g^-1^ |
| pH | 7.6 | - |
| Bulk density | 1.65 | g cm^-3^ |
| Particle density | 2.83 | g cm^-3^ |
| Porosity | 0.41 | cm^-3^ cm^-3^ |
| Moisture content | 0.086 | % |
| **Mineralogy** |  |  |
| Quartz | 19.74 | % |
| Plagioclase | 39.49 | % |
| Biotite/Fe Illite | 10.60 | % |
| Fe-Ox/CO3 | 4.84 | % |
| Mg Fe silicates | 4.83 | % |
| Others | 8.89 | % |
| ^1^ - Tailings samples (<2 mm) were analysed for particle size using a laser diffraction analyser (Mastersizer 3000 with the HydroLV attachment; Malvern Panalytical). Bulk density was measured by collecting a known volume of tailings using a core and determining the mass after oven-drying. Particle density was determined according to BS 1377: Part 2: 1990 using a pycnometer. Porosity was calculated from bulk density and particle density. The pH was determined by mixing them with deionised water at a 1:10 solid:liquid ratio (m v^-1^) and taking a pH reading after 4 hours. Cation Exchange Capacity (CEC) was determined by soaking tailings with a 1 N ammonium acetate solution (pH 7) for 24 hours and vacuum filtrated washing with a solution of 1 N ammonium chloride. The material was dried with 50 mL of deionised water added, and 0.5 mL of NaOH was then added to release ammonium. CEC was calculated using the concentration of released ammonium via an ammonium ion-selective electrode according to ISO/TS 22171. Mineralogy determined using Quantitative Evaluation of Minerals by Scanning Electron Microscopy (QEMSCAN) comprising an area of area of 27 mm^2^ at 10 µm X-ray pixel spacing. | | |

| **Table S2** – Metal recovery (%) based on the total elemental concentration in tailings prior leaching with different LMWOAs at 1 M (or water as control), and the concentration in leachates after solvent application (50 mL). Values are averages (n = 4) with standard errors. Percentages were calculated for 200 g of treated tailings. Solvents were applied at different dilution rates: ¼ (250 mM), 1/16 (62 mM) and 1/128 (8 mM). | | | | | | | | | | |
| --- | --- | --- | --- | --- | --- | --- | --- | --- | --- | --- |
|  | **Cu %** | **Al %** | **Mg %** | **K %** | **Ca %** | **Mn %** | **Fe %** | **Zn %** | **V %** |  |
| **Control** | < 0.01 | < 0.01 | 0.2 ± 0.02 | 0.4 ± 0.01 | 0.7 ± 0.07 | < 0.01 | < 0.01 | < 0.01 | < 0.01 |  |
| **Dilution 1/4** |  |  |  |  |  |  |  |  |  |  |
| Citric | 10.9 ± 1.7 | 13.0 ± 0.4 | 4.4 ± 0.1 | 1.8 ± 0.04 | 10.1 ± 0.1 | 3.0 ± 0.1 | 28.3 ± 0.7 | 0.08 ± 0.01 | < 0.01 |  |
| Malic | 16.8 ± 1.1 | 7.6 ± 0.4 | 2.7 ± 0.2 | 1.5 ± 0.1 | 7.9 ± 0.3 | 2.2 ± 0.2 | 20.7 ± 1.4 | 0.07 ± 0.01 | < 0.01 |  |
| Maleic | 8.4 ± 0.8 | 1.2 ± 0.2 | 3.3 ± 0.3 | 2.6 ± 0.2 | 13.6 ± 1.1 | 1.7 ± 0.2 | 0.2 ± 0.03 | 0.06 ± 0.003 | < 0.01 |  |
| Malonic | 11.8 ± 0.2 | 8.8 ± 0.2 | 2.3 ± 0.1 | 1.6 ± 0.02 | 8.0 ± 0.03 | 1.2 ± 0.03 | 6.0 ± 0.2 | 0.03 ± 0.001 | 0.02 ± 0.001 |  |
| -Lactic | 11.6 ± 0.7 | 3.5 ± 0.2 | 2.1 ± 0.1 | 1.7 ± 0.1 | 8.3 ± 0.4 | 1.9 ± 0.1 | 9.4 ± 0.5 | 0.1 ± 0.02 | < 0.01 |  |
| Citric + Malic | 9.2 ± 1.9 | 11.1 ± 0.6 | 4.5 ± 0.3 | 2.2 ± 0.1 | 11.6 ± 0.5 | 3.4 ± 0.1 | 30.3 ± 1.3 | 0.1 ± 0.002 | < 0.01 |  |
|  |  |  |  |  |  |  |  |  |  |  |
| **Dilution 1/16** |  |  |  |  |  |  |  |  |  |  |
| Citric | 3.8 ± 0.3 | 2.9 ± 0.3 | 1.1 ± 0.1 | 0.9 ± 0.08 | 4.7 ± 0.4 | 0.4 ± 0.08 | 6.4 ± 0.5 | < 0.01 | < 0.01 |  |
| Malic | 3.1 ± 0.3 | 1.1 ± 0.1 | 0.7 ± 0.02 | 0.7 ± 0.02 | 3.2 ± 0.2 | 0.1 ± 0.02 | 2.1 ± 0.4 | < 0.01 | < 0.01 |  |
| Maleic | 0.6 ± 0.1 | < 0.01 | 0.9 ± 0.03 | 1.1 ± 0.04 | 4.0 ± 0.2 | 0.1 ± 0.01 | 0.03 ± 0.01 | < 0.01 | < 0.01 |  |
| Malonic | 2.9 ± 0.2 | 0.8 ± 0.2 | 0.8 ± 0.04 | 0.7 ± 0.05 | 3.2 ± 0.2 | 0.1 ± 0.01 | 0.04 ± 0.02 | < 0.01 | < 0.01 |  |
| Lactic | 0.2 ± 0.1 | 0.01 ± 0.01 | 0.9 ± 0.01 | 1.1 ± 0.01 | 4.1 ± 0.1 | 0.3 ± 0.05 | 0.07 ± 0.02 | < 0.01 | < 0.01 |  |
| Citric + Malic | 2.0 ± 0.4 | 2.3 ± 0.1 | 1.1 ± 0.1 | 1.2 ± 0.07 | 5.0 ± 0.2 | 0.5 ± 0.09 | 5.1 ± 0.7 | < 0.01 | < 0.01 |  |
|  |  |  |  |  |  |  |  |  |  |  |
| **Dilution 1/128** |  |  |  |  |  |  |  |  |  |  |
| Citric | 0.1 ± 0.02 | < 0.01 | 0.3 ± 0.01 | 0.6 ± 0.02 | 1.1 ± 0.03 | < 0.01 | 0.05 ± 0.01 | < 0.01 | < 0.01 |  |
| Malic | 0.01 ± 0.00 | < 0.01 | 0.3 ± 0.01 | 0.6 ± 0.01 | 1.1 ± 0.04 | < 0.01 | 0.01 ± 0.01 | < 0.01 | < 0.01 |  |
| Maleic | < 0.01 | < 0.01 | 0.3 ± 0.01 | 0.6 ± 0.01 | 1.1 ± 0.08 | < 0.01 | < 0.01 | < 0.01 | < 0.01 |  |
| Malonic | < 0.01 | < 0.01 | 0.2 ± 0.01 | 0.5 ± 0.02 | 0.9 ± 0.06 | < 0.01 | < 0.01 | < 0.01 | < 0.01 |  |
| Lactic | < 0.01 | < 0.01 | 0.3 ± 0.02 | 0.5 ± 0.02 | 1.1 ± 0.07 | 0.06 ± 0.01 | < 0.01 | < 0.01 | < 0.01 |  |
| Citric + Malic | < 0.01 | < 0.01 | 0.2 ± 0.01 | 0.5 ± 0.02 | 0.8 ± 0.05 | 0.02 ± 0.01 | < 0.01 | < 0.01 | < 0.01 |  |
